# Supplementary material for: Resampling-based methods for biologists
Source: PeerJ. 2020 May 7;8:e9089. doi: 10.7717/peerj.9089 (PMC7211410; doi:10.7717/peerj.9089)
Supplement: Supplemental Information 1 — This simulation example demonstrates how to conduct a permutation-based test for a partial regression coefficient in a multiple linear regression model. [file peerj-08-9089-s001.pdf]

Supplementary Appendix A: Linear Regression Example

jfieberg

2020-01-09

Objective

This simulation example demonstrates how to conduct a permutation-based test for a partial regression coefficient in a multiple linear regression model.

Document Preamble

```
# Load Libraries
library(knitr)
library(mosaic)
library(ggplot2)
library(MASS)

# Set knitr options
opts_chunk$set(fig.width = 6, fig.height=5)

# Clear Environment (optional)
remove(list=ls())

# Set seed
set.seed(314159)
```

Simulation Example

Here we will consider a simple simulation where a response variable, y, is related to two predictor variables, x1 and x2. The predictors are themselves correlated. We will illustrate a simple permutation-based test for the effect of x1, adjusted for x2.

Steps:

1. Fit a linear regression model relating x1 to x2.
2. Add the residuals from this model to the original data set.
3. Create the permutation distribution by shuffling these residuals.
4. Determine the p-value by comparing the t-statistic from the fit to the original data set to the permutation-based distribution of this same statistic.

Simulation parameters

- Sigma (variance/covariance matrix of x1 and x2).
- We will assume mean of x1 and x2 =0
- Beta = vector of regression parameters (with intercept=0)

```
Sigma <- matrix(c(10,3,3,2),2,2)
Beta <- c(0.2, -0.5)
```

Create correlated predictors

```
X<- mvrnorm(n = 100, rep(0, 2), Sigma)
cor(X)
```

```
##             [,1]      [,2]
## [1,] 1.0000000 0.6054432
## [2,] 0.6054432 1.0000000
```

Form response variables

```
y<-X%*%Beta+rnorm(100,0,2)
Mydata<-data.frame(y=y, x1=X[,1], x2=X[,2])
```

Fit regression model to the data

```
lmsim<-lm(y~x1+x2, data=Mydata)
summary(lmsim)
```

```
##
## Call:
## lm(formula = y ~ x1 + x2, data = Mydata)
##
## Residuals:
##     Min      1Q  Median      3Q     Max
## -4.9605 -1.3618 -0.1088  1.2206  5.3751
##
## Coefficients:
##             Estimate Std. Error t value Pr(>|t|)
## (Intercept) -0.05740    0.21377  -0.269  0.78888
## x1           0.18488    0.08781   2.105  0.03783 *
## x2          -0.66629    0.20420  -3.263  0.00152 **
## ---
## Signif. codes:  0 '***' 0.001 '**' 0.01 '*' 0.05 '.' 0.1 ' ' 1
##
## Residual standard error: 2.134 on 97 degrees of freedom
## Multiple R-squared:  0.09913,    Adjusted R-squared:  0.08056
## F-statistic: 5.337 on 2 and 97 DF,  p-value: 0.006326
```

Step 1: capture the part of x1 that is not related to x2

```
lm1<-lm(x1~x2, data=Mydata)
Mydata<-Mydata %>% mutate(x1resid=lm1$resid)
```

Demonstrate that using the residuals here results in the same coefficient, standard error, t-statistic and p-value for x1 as in our original regression (lmsim)

```
lmsim2<-lm(y~x1resid+x2, data=Mydata)
summary(lmsim2)
```

```
##
## Call:
## lm(formula = y ~ x1resid + x2, data = Mydata)
##
## Residuals:
##     Min      1Q  Median      3Q     Max
## -4.9605 -1.3618 -0.1088  1.2206  5.3751
##
## Coefficients:
##             Estimate Std. Error t value Pr(>|t|)
## (Intercept) -0.04442    0.21368  -0.208   0.8358
## x1resid      0.18488    0.08781   2.105   0.0378 *
## x2          -0.40599    0.16252  -2.498   0.0142 *
```

```
## ---
## Signif. codes:  0 '***' 0.001 '**' 0.01 '*' 0.05 '.' 0.1 ' ' 1
##
## Residual standard error: 2.134 on 97 degrees of freedom
## Multiple R-squared:  0.09913,    Adjusted R-squared:  0.08056
## F-statistic: 5.337 on 2 and 97 DF,  p-value: 0.006326
```

Store the t-statistic for x1 from this model

```
(tstat<-summary(lmsim)$coefficients[2,3])
```

```
## [1] 2.105456
```

Step 2: create the permutation distribution

```
randsims<-do(10000)*{
  lmrand<-lm(y~shuffle(x1resid)+x2, data=Mydata)
  summary(lmrand)$coefficients[2,3]
}
head(randsims)
```

```
##       result
## 1 -0.3195885
## 2  1.6825919
## 3  1.2424042
## 4 -0.5098583
## 5 -0.5584336
## 6  0.4883965
```

Plot the randomization distribution with our original statistic

```
histogram(~result, data=randsims, v=tstat, col="gray")
```

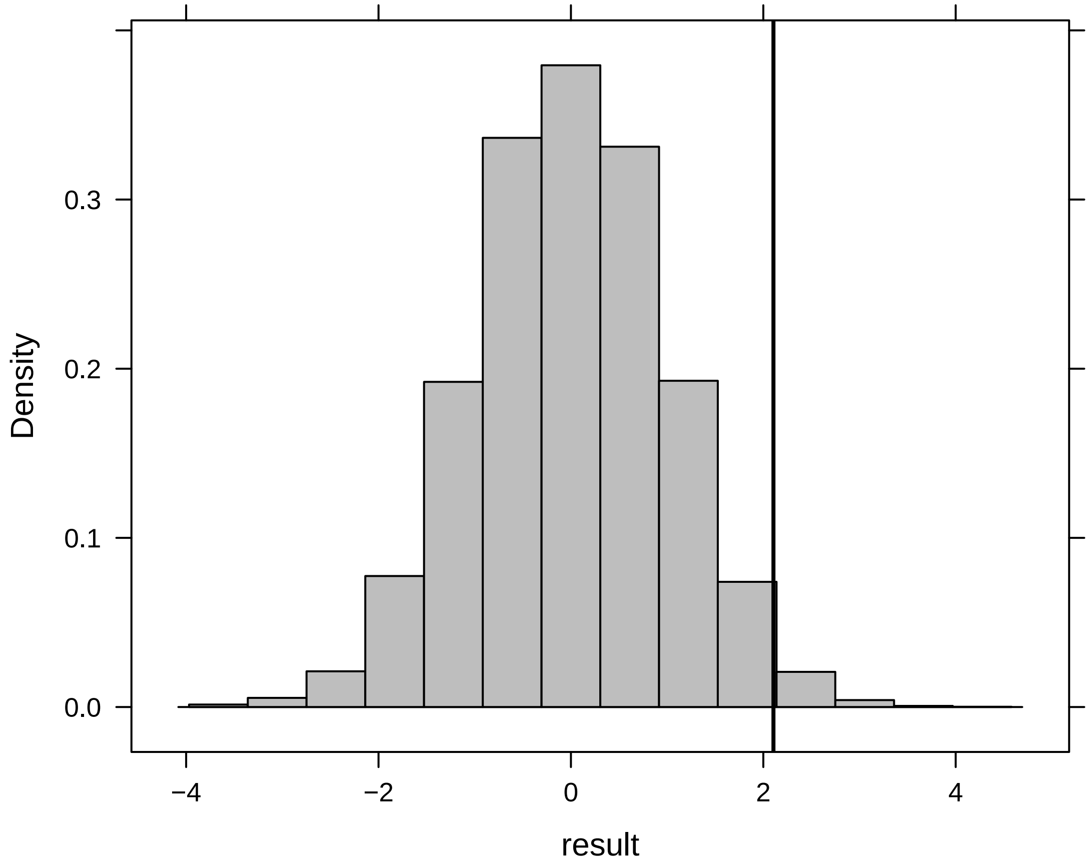

Determine our p-value

```
prop(~I(abs(result)>=tstat), data=randsims)
```

```
## prop_TRUE
##     0.036
```

Conclusions

The permutation-based approach allows us to relax the Normality assumption. Our randomization-based p-value is really similar to the p-value of the original t-test. This result is not surprising given that the assumptions of linear regression (constant variance, normality, linearity) all hold in the simulation example.

Document footer

Session Information:

```
sessionInfo()
```

```
## R version 3.6.1 (2019-07-05)
## Platform: x86_64-w64-mingw32/x64 (64-bit)
## Running under: Windows 10 x64 (build 17763)
##
## Matrix products: default
##
```

```
## Random number generation:
##  RNG:     Mersenne-Twister
##  Normal:  Inversion
##  Sample:  Rounding
##
## locale:
## [1] LC_COLLATE=English_United States.1252
## [2] LC_CTYPE=English_United States.1252
## [3] LC_MONETARY=English_United States.1252
## [4] LC_NUMERIC=C
## [5] LC_TIME=English_United States.1252
##
## attached base packages:
## [1] splines   stats     graphics  grDevices utils     datasets  methods
## [8] base
##
## other attached packages:
##  [1] MASS_7.3-51.4     rms_5.1-3.1       SparseM_1.77
##  [4] Hmisc_4.2-0       Formula_1.2-3     survival_2.44-1.1
##  [7] mgcv_1.8-28       nlme_3.1-140      gmodels_2.18.1
## [10] geepack_1.2-1     boot_1.3-22       ggfortify_0.4.7
## [13] mosaic_1.5.0      Matrix_1.2-17     mosaicData_0.17.0
## [16] ggformula_0.9.2   ggstance_0.3.3    ggplot2_3.2.1
## [19] lattice_0.20-38   dplyr_0.8.3       knitr_1.25
##
## loaded via a namespace (and not attached):
##  [1] RColorBrewer_1.1-2  tools_3.6.1         backports_1.1.5
##  [4] utf8_1.1.4          R6_2.4.0            rpart_4.1-15
##  [7] lazyeval_0.2.2      colorspace_1.4-1    nnet_7.3-12
## [10] withr_2.1.2         tidyselect_0.2.5    gridExtra_2.3
## [13] leaflet_2.0.2       compiler_3.6.1      quantreg_5.51
## [16] cli_1.1.0           htmlTable_1.13.2    sandwich_2.5-1
## [19] ggdendro_0.1-20     labeling_0.3        mosaicCore_0.6.0
## [22] scales_1.0.0        checkmate_1.9.4     mvtnorm_1.0-11
## [25] polspline_1.1.16    readr_1.3.1         stringr_1.4.0
## [28] digest_0.6.22       foreign_0.8-71      rmarkdown_1.18
## [31] base64enc_0.1-3     pkgconfig_2.0.3     htmltools_0.4.0
## [34] fastmap_1.0.1       highr_0.8           htmlwidgets_1.5.1
## [37] rlang_0.4.1         rstudioapi_0.10     shiny_1.4.0
## [40] generics_0.0.2      zoo_1.8-6           crosstalk_1.0.0
## [43] gtools_3.8.1        acepack_1.4.1       magrittr_1.5
## [46] Rcpp_1.0.2          munsell_0.5.0       fansi_0.4.0
## [49] lifecycle_0.1.0     multcomp_1.4-10     stringi_1.4.3
## [52] yaml_2.2.0          grid_3.6.1          gdata_2.18.0
## [55] promises_1.1.0      ggrepel_0.8.1       crayon_1.3.4
## [58] hms_0.5.2           zeallot_0.1.0       pillar_1.4.2
## [61] codetools_0.2-16    glue_1.3.1          packrat_0.5.0
## [64] evaluate_0.14       latticeExtra_0.6-28 data.table_1.12.6
## [67] vctrs_0.2.0         httpuv_1.5.2        MatrixModels_0.4-1
## [70] gtable_0.3.0        purrr_0.3.3         tidyr_1.0.0
## [73] assertthat_0.2.1    xfun_0.10           mime_0.7
## [76] xtable_1.8-4        broom_0.5.2         later_1.0.0
## [79] tibble_2.1.3        tinytex_0.17        cluster_2.1.0
## [82] TH.data_1.0-10
```
