# Supplementary material for: Resampling-based methods for biologists
Source: PeerJ. 2020 May 7;8:e9089. doi: 10.7717/peerj.9089 (PMC7211410; doi:10.7717/peerj.9089)
Supplement: Supplemental Information 2 — This example demonstrates how: (1) a cluster-level bootstrap can be used for repeated measures data with equal-sized clusters. This approach is most applicable to problems where predictors of interest do not vary within a cluster (2) to use functions in the boot package to calculate different bootstrap confidence intervals, including the BCa interval, which has better statistical properties than percentile-based intervals. [file peerj-08-9089-s002.pdf]

Case Study I: Relaxing the Assumptions of Linear Regression

jfieberg

2020-01-09

Objectives

This example demonstrates how:

1. a cluster-level bootstrap can be used for repeated measures data with equal-sized clusters. This approach is most applicable to problems where predictors of interest do not vary within a cluster.
2. to use functions in the boot package to calculate different bootstrap confidence intervals, including the BCa interval, which has better statistical properties than percentile-based intervals.

Document Preamble

```
# Load Libraries
 library(knitr)
 library(mosaic)
 library(ggplot2)
 library(ggfortify)

# Set knitr options
opts_chunk$set(fig.width = 6, fig.height=5)

# Clear Environment (optional)
remove(list=ls())

# Set seed
set.seed(314159)
```

Section 1. Bootstrapping RIKZ data

Read in data from .csv and look at first 6 rows

```
rikzData <- read.csv("data/RIKZdat.csv")
head(rikzData)
```

```
##   week angle1 angle2 exposure salinity temperature    NAP penetrability
## 1    1     32     96       10     29.4        17.5  0.045         253.9
## 2    1     62     96       10     29.4        17.5 -1.036         226.9
## 3    1     65     96       10     29.4        17.5 -1.336         237.1
## 4    1     55     96       10     29.4        17.5  0.616         248.6
## 5    1     23     96       10     29.4        17.5 -0.684         251.9
## 6    1    129     89        8     29.6        20.8  1.190         250.1
##   grainsize humus chalk sorting1 Beach Richness
## 1     222.5  0.05  2.05   69.830     1       11
## 2     200.0  0.30  2.50   59.000     1       10
## 3     194.5  0.10  3.45   59.220     1       13
## 4     221.0  0.15  1.60   67.750     1       11
## 5     202.0  0.05  2.45   57.760     1       10
```

```
## 6     192.5  0.10  2.50   53.075     2        8
```

Fit a linear regression model relating species richness to exposure level

```r
# Simple linear regression and summary
lm.RIKZ <- lm(Richness~exposure, data=rikzData)
summary(lm.RIKZ)
```

```
##
## Call:
## lm(formula = Richness ~ exposure, data = rikzData)
##
## Residuals:
##     Min      1Q  Median      3Q     Max
## -6.3882 -2.2412 -0.2412  1.7588 15.6118
##
## Coefficients:
##             Estimate Std. Error t value Pr(>|t|)
## (Intercept)  37.8588     6.8682   5.512 1.86e-06 ***
## exposure     -3.1471     0.6692  -4.703 2.66e-05 ***
## ---
## Signif. codes:  0 '***' 0.001 '**' 0.01 '*' 0.05 '.' 0.1 ' ' 1
##
## Residual standard error: 4.113 on 43 degrees of freedom
## Multiple R-squared:  0.3396, Adjusted R-squared:  0.3243
## F-statistic: 22.11 on 1 and 43 DF,  p-value: 2.664e-05
```

Check Assumptions

Here, we see that the residuals do not appear Normally distributed. We also know the independence assumption is problematic.

```r
par(mfrow=c(1,2))
with(rikzData, plot(exposure, Richness, pch=16, bty="L", cex.lab=1.4))
abline(lm.RIKZ)
title("A)", adj=0)
xdat<-range(lm.RIKZ$resid)
hist(lm.RIKZ$resid, col="gray", xlab="Residuals", freq=FALSE, bty="L", cex.lab=1.4, main="")
title("B)", adj=0)
curve(dnorm(x, mean(lm.RIKZ$resid), sd(lm.RIKZ$resid)), from =xdat[1], to=xdat[2],
      add=TRUE, col="black")
```

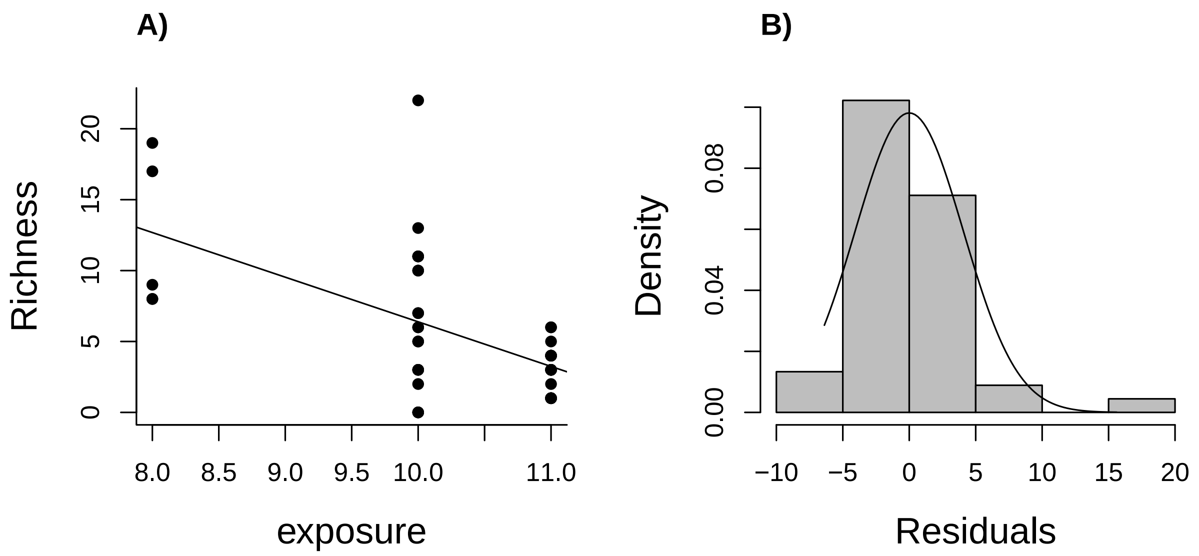

Cluster-level bootstrap example. The code below shows how to create a single bootstrap data set where we resample clusters.

```
# Data processing
uid <- unique(rikzData$Beach) # unique id for each beach
nBeach <- length(uid) # number of beaches

### One bootstrap:
# Take a sample from x (uid) of size nBeach with replacement:
bootIDs <- data.frame(Beach = sample(x = uid, size = nBeach, replace = TRUE))
bootIDs
```

```
##   Beach
## 1     2
## 2     6
## 3     3
## 4     4
## 5     9
## 6     5
## 7     6
## 8     3
## 9     4
```

```
# Use this to sample from original data by beach
bootDat <- merge(bootIDs, rikzData)
table(bootDat$Beach) ## this table shows how many obs are drawn for each beach in bootstrap sample.
```

```
##
##  2  3  4  5  6  9
##  5 10 10  5 10  5
```

```
# Double check sample sizes worked (these should match):
length(rikzData$Beach) # original data
```

```
## [1] 45
```

```r
length(bootDat$Beach) # bootstrap sample
```

```
## [1] 45
```

Now, repeat this process several times and store results.

```r
nBoots <- 5000 # number of bootstraps
coef.ests <- matrix(NA, ncol=2, nrow=nBoots) # to hold bootstrap estimates
for(i in 1:nBoots){
  # create bootstrap
  bootIDs <- data.frame(Beach = sample(x = uid, size = nBeach, replace = TRUE))
  bootDat <- merge(bootIDs, rikzData)

  # Estimate coefficients
  lmBoot <- lm(Richness ~ exposure, data=bootDat)
  coef.ests[i,] <- lmBoot$coefficients
}

# Look at histograms of the bootstrap estimates
par(mfrow=c(1,2))
hist(coef.ests[,1], main="", xlab="intercept")
hist(coef.ests[,2], main="", xlab="slope")
```

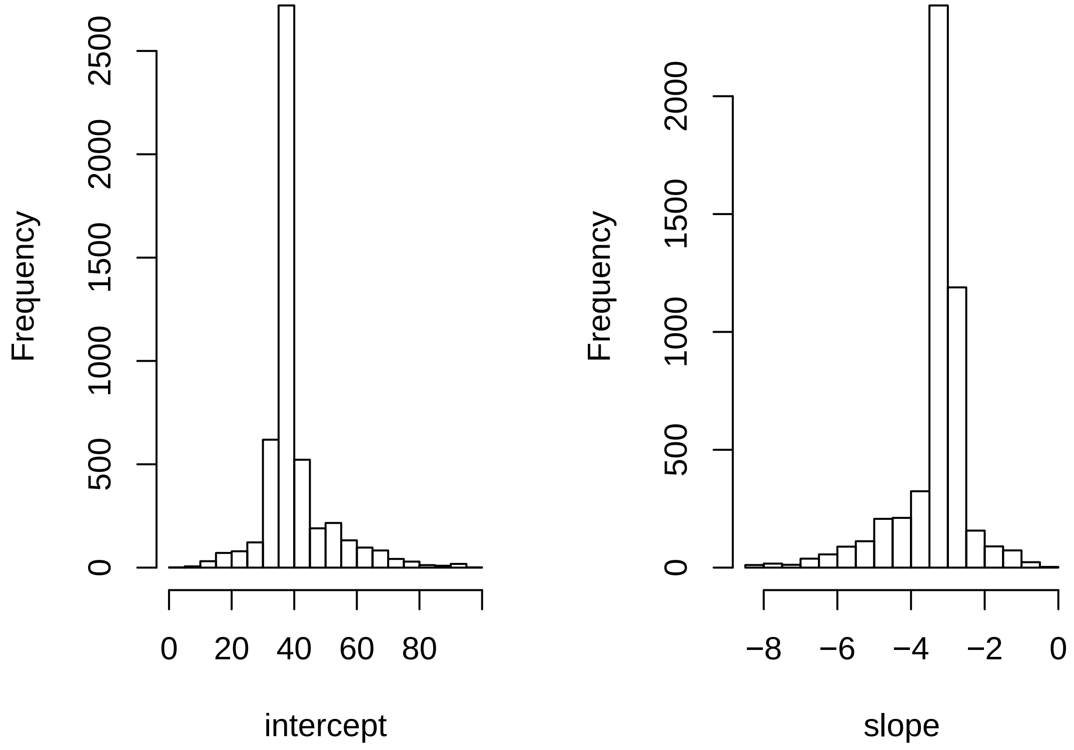

```r
# Bootstrap confidence intervals using the percentile method
# Intercept:
quantile(x = coef.ests[,1], probs = c(0.025,0.975), na.rm=TRUE)
```

```
##     2.5%    97.5%
## 21.44875 69.00000
```

```r
# Slope:
quantile(x = coef.ests[,2], probs = c(0.025,0.975), na.rm=TRUE)
```

```
##   2.5%  97.5%
## -6.003 -1.680
```

```r
# Compare to linear regression confidence intervals:
confint(lm.RIKZ)
```

```
##                 2.5 %    97.5 %
## (Intercept) 24.007705 51.709942
## exposure    -4.496649 -1.797469
```

Better bootstrap confidence intervals

Note: Although it is common to use percentile-based bootstrap confidence intervals, there are better ways to calculate confidence intervals, particularly when the bootstrap distribution is not symmetric (as is the case here). A nice discussion of alternative bootstrap confidence intervals is Hesterberg (2015). What Teachers Should Know about the Bootstrap: Resampling in the Undergraduate Statistics Curriculum, The American Statistician 69:371-386.

Below, I illustrate how one can use the boot library to calculate some alternative intervals.

Other alternatives using boot.ci in the boot library

- Normal = estimate +/- 1.96*SE(bootstrap distribution)
- Percentile = percentile-based
- Basic = calculates differences between theta[boot] and original estimate and uses this information to construct the interval
- BCa = attempts to correct for bias and skewness in the bootstrap distribution.

Load boot library

```r
library(boot)
```

To use the functions in the boot library, we have to create a function with first two arguments = data, indices

- data = the data that will be resampled
- indices are the vector of indices that will be resampled

```r
slope.exposure<-function(data, indices, formula, obsdat){
    # data will contain the data to be resampled (here, beach ids)
    # indices = used to select clusters
    # formula = allows flexibility w/ the model that is fit
    # obsdat = our data set containing all observations

    bootbeaches<-data[indices]
    bootids<-data.frame(Beach=bootbeaches)
    bootdat<-merge(bootids, obsdat)

# Fit the linear model with exposurec and return the fitted coefficients
   lm.boot<- lm(formula, data = bootdat)
```

```r
  return(coef(lm.boot))
}

# Call the function to do the bootstrapping
results<-boot(data=uid, statistic = slope.exposure,  R=9999,
              formula=Richness ~ exposure, obsdat=rikzData)
```

Lets look at the results. Note: the warning, below, tells us that we are unable to calculate studentized intervals (these are discussed in Hesterberg (2015)) and tend to work well in many situations but require an estimate of variance of the statistic to go along with each bootstrap replicate. Nonetheless, we get several other intervals all from the same set of bootstrapped coefficients. Of these, the BCa interval is arguably the most defensible approach.

```r
# Look at results: Intercept
boot.ci(results, index=1)
```

```
## Warning in boot.ci(results, index = 1): bootstrap variances needed for
## studentized intervals
```

```
## BOOTSTRAP CONFIDENCE INTERVAL CALCULATIONS
## Based on 9999 bootstrap replicates
##
## CALL :
## boot.ci(boot.out = results, index = 1)
##
## Intervals :
## Level      Normal              Basic
## 95%   (15.70, 56.33 )   ( 6.82, 55.62 )
##
## Level     Percentile            BCa
## 95%   (20.10, 68.90 )   (27.80, 78.47 )
## Calculations and Intervals on Original Scale
```

```r
# Look at results: Slope
boot.ci(results, index=2)
```

```
## Warning in boot.ci(results, index = 2): bootstrap variances needed for
## studentized intervals
```

```
## BOOTSTRAP CONFIDENCE INTERVAL CALCULATIONS
## Based on 9992 bootstrap replicates
##
## CALL :
## boot.ci(boot.out = results, index = 2)
##
## Intervals :
## Level      Normal              Basic
## 95%   (-4.833, -1.111 )   (-4.715, -0.294 )
##
## Level     Percentile            BCa
## 95%   (-6.000, -1.579 )   (-6.600, -2.033 )
## Calculations and Intervals on Original Scale
```

The percentile-based intervals are very similar to the intervals we calculated previously. Note, however the BCA interval (preferred) is highly asymmetric. All intervals are quite a bit wider than those calculated assuming independence. Lets plot the results for a visual comparison using ggplot.

```r
library(ggplot2)
```

Gather the data.

```r
intervals<-matrix(NA,10,2)

# Interepts
intervals[1,]<-confint(lm.RIKZ)[1,] # CI assuming independence
intervals[2,]<- boot.ci(results, index=1)$normal[2:3] #normal based
intervals[3,]<- boot.ci(results, index=1)$basic[4:5] # basic
intervals[4,]<- boot.ci(results, index=1)$percent[4:5] # percentile
intervals[5,]<- boot.ci(results, index=1)$bca[4:5] # bias-corrected and accelerated

# Slopes
intervals[6,]<-confint(lm.RIKZ)[2,] # CI assuming independence
intervals[7,]<- boot.ci(results, index=2)$normal[2:3] # normal
intervals[8,]<- boot.ci(results, index=2)$basic[4:5] #basic
intervals[9,]<- boot.ci(results, index=2)$percent[4:5] #percentil
intervals[10,]<- boot.ci(results, index=2)$bca[4:5]
```

Reformat the data for plotting

```r
intervals.df<-data.frame(lowerCL=intervals[,1], upperCL=intervals[,2],
                         parameter=c(rep("Intercept",5), rep("Slope",5)),
                         type=rep(c("Independence", "Normal", "Basic", "Percentile", "BCa"), 2),
                         PE=rep(coef(lm.RIKZ), each=5))
```

Plot

```r
  ggplot(intervals.df, aes(x=type, y=PE, group=type)) +
    geom_errorbar(width=.1, aes(ymin=lowerCL, ymax=upperCL), colour="blue") +
    geom_point(shape=21, size=3, fill="white")+
    facet_wrap(~parameter, ncol=2, scales="free") + xlab("")+ylab("Estimate and 95% CI")
```

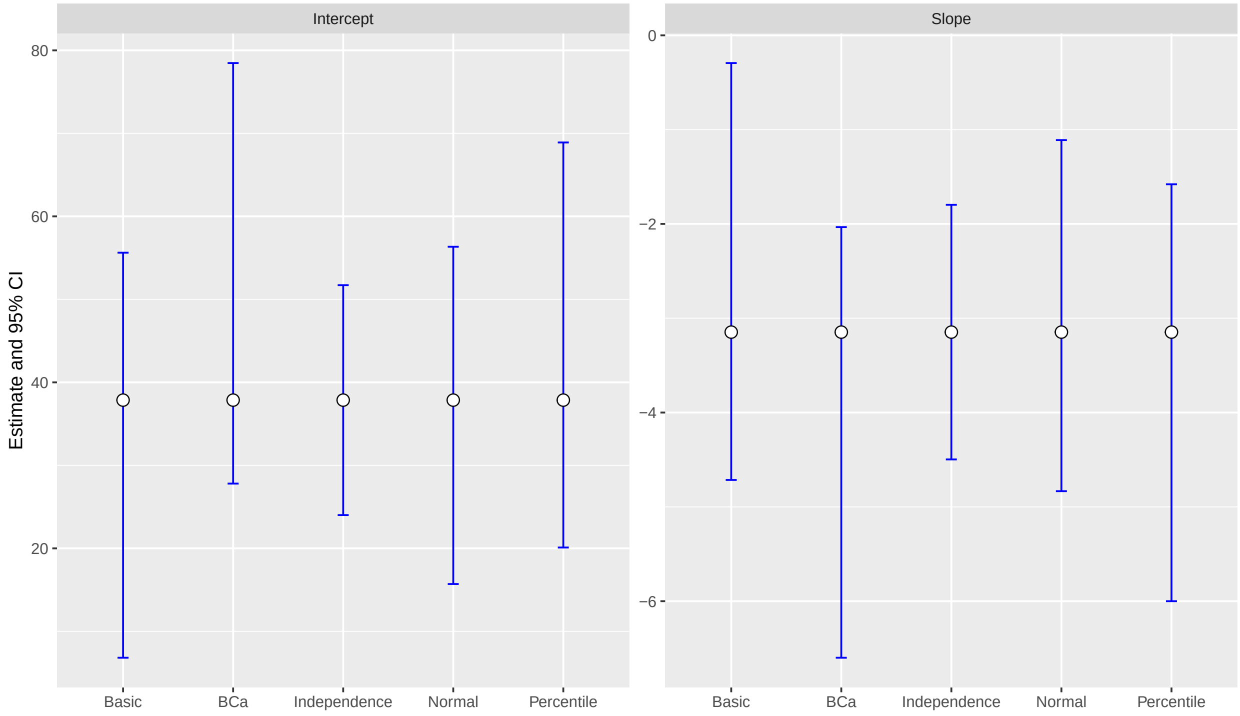

Conclusions

Using a cluster-level bootstrap results in wider confidence intervals than naive intervals that assume independence. Also, the BCa intervals, which have better statistical properties than percentile-based intervals when estimators exhibit bias or the sampling distribution is skewed, result is a highly asymmetric confidence interval.

Document footer

Session Information:

```
sessionInfo()
```

```
## R version 3.6.1 (2019-07-05)
## Platform: x86_64-w64-mingw32/x64 (64-bit)
## Running under: Windows 10 x64 (build 17763)
##
## Matrix products: default
##
## Random number generation:
##  RNG:     Mersenne-Twister
##  Normal:  Inversion
##  Sample:  Rounding
##
## locale:
## [1] LC_COLLATE=English_United States.1252
## [2] LC_CTYPE=English_United States.1252
## [3] LC_MONETARY=English_United States.1252
## [4] LC_NUMERIC=C
## [5] LC_TIME=English_United States.1252
```

```
##
## attached base packages:
## [1] stats     graphics  grDevices utils     datasets  methods   base
##
## other attached packages:
##  [1] boot_1.3-22       ggfortify_0.4.7   mosaic_1.5.0
##  [4] Matrix_1.2-17     mosaicData_0.17.0 ggformula_0.9.2
##  [7] ggstance_0.3.3    ggplot2_3.2.1     lattice_0.20-38
## [10] dplyr_0.8.3       knitr_1.25
##
## loaded via a namespace (and not attached):
##  [1] tidyselect_0.2.5  xfun_0.10         purrr_0.3.3
##  [4] splines_3.6.1     colorspace_1.4-1  vctrs_0.2.0
##  [7] generics_0.0.2    htmltools_0.4.0   yaml_2.2.0
## [10] rlang_0.4.1       pillar_1.4.2      later_1.0.0
## [13] glue_1.3.1        withr_2.1.2       lifecycle_0.1.0
## [16] mosaicCore_0.6.0  stringr_1.4.0     munsell_0.5.0
## [19] gtable_0.3.0      htmlwidgets_1.5.1 evaluate_0.14
## [22] labeling_0.3      fastmap_1.0.1     httpuv_1.5.2
## [25] crosstalk_1.0.0   highr_0.8         broom_0.5.2
## [28] Rcpp_1.0.2        readr_1.3.1       xtable_1.8-4
## [31] scales_1.0.0      backports_1.1.5   promises_1.1.0
## [34] leaflet_2.0.2     mime_0.7          gridExtra_2.3
## [37] hms_0.5.2         packrat_0.5.0     digest_0.6.22
## [40] stringi_1.4.3     ggrepel_0.8.1     shiny_1.4.0
## [43] grid_3.6.1        tools_3.6.1       magrittr_1.5
## [46] lazyeval_0.2.2    tibble_2.1.3      ggdendro_0.1-20
## [49] crayon_1.3.4      tidyr_1.0.0       pkgconfig_2.0.3
## [52] zeallot_0.1.0     MASS_7.3-51.4     assertthat_0.2.1
## [55] rmarkdown_1.18    R6_2.4.0          nlme_3.1-140
## [58] compiler_3.6.1
```
