# Supplementary material for: Resampling-based methods for biologists
Source: PeerJ. 2020 May 7;8:e9089. doi: 10.7717/peerj.9089 (PMC7211410; doi:10.7717/peerj.9089)
Supplement: Supplemental Information 3 — This example demonstrates how: (1) the bootstrap can be used in applications that involve multiple response measures from the same set of cases (2) the bootstrap can provide estimates of uncertainty for non-linear functions of model parameters. [file peerj-08-9089-s003.pdf]

Case Study II: Combining results from multiple models

jfieberg

2020-01-09

Objectives

This example demonstrates how:

1. the bootstrap can be used in applications that involve multiple response measures from the same set of cases.
2. the bootstrap can provide estimates of uncertainty for non-linear functions of model parameters. In such cases, there will usually not be an analytical formula for calculating the SE of our estimator. Alternatives are to use the delta method with a Normal approximation of the sampling distribution or Bayesian methods.

This example comes from:

Zicus, M. C., D. P. Rave, and J. Fieberg. 2006. Cost effectiveness of single- vs. double-cylinder over-water nest structures. Wildlife Society Bulletin 34:647-655.

```
library(geepack)
library(gmodels)
library(mgcv)
library(splines)
library(dplyr)
library(ggplot2)
library(ggfortify)
```

Set seed of random number generator

```
set.seed(10)
```

Read in survival data and duckling data

```
  ddata<-read.csv("data/costeff.csv")
  names(ddata)<-tolower(names(ddata))
  ddata$deply<-as.factor(ddata$deply)
  ddata$year<-as.factor(ddata$year)
```

Variables of interest:

- deply = 0 for single cylinders and 1 for double cylinders
- strtno = structure ID (unique to each nesting structure)
- period = (1-4) categorical variable capturing seasonal effects
- size = size of the wetland where the structure is placed
- year = year of observation
- yng = number of ducks produced

Make sure the observations are ordered by structure ID (strtno);

```
  x<-order(ddata$strtno)
  ddata<-ddata[x,]
```

Structure survival model

Place knots at size = 3 and size = 10 and create spline basis vectors when modeling the (non-linear) effect of wetland size

```
bsize<-ns(ddata$size,df=3, knots=c(3,10))
ddata2<-cbind(ddata,bsize[,1:3])
names(ddata2)[8:10]<-c("sz1", "sz2", "sz3")
```

Fit the discrete time survival model to capture influence of structure type (deply) and wetland size.

```
glmsurv<-glm(surv~year+deply +sz1+sz2+sz3, family=binomial(link=cloglog), data=ddata2)
```

Now, lets get predictions for both cylinder types, a range of sizes, and all 8 years.

```
pdat<-expand.grid(size = seq(0,28,0.1), deply=unique(ddata2$deply), year=unique(ddata2$year))
```

Create the spline basis vectors again, using the same set of knots

```
pdatsize<-predict(bsize, pdat$size)
pdat2<-cbind(pdat,pdatsize[,1:3])
names(pdat2)[4:6]<-c("sz1", "sz2", "sz3")
```

Now, predictions on prob of failure (accounting for the fact that our model is fit on the cloglog scale)

```
pdat2$pfail<-predict(glmsurv, newdata=pdat2, type="resp")
```

Plot survival for each year and deployment type. We see that structure failure rates increase with the size of the wetland. These failure rates also varied considerably from year to year.

```
ggplot(pdat2, aes(x=size, y=pfail, color=year))+geom_line()+facet_wrap(~deply)+
 xlab("Size of wetland (ha)")+ ylab("Probability of Structure Failure")
```

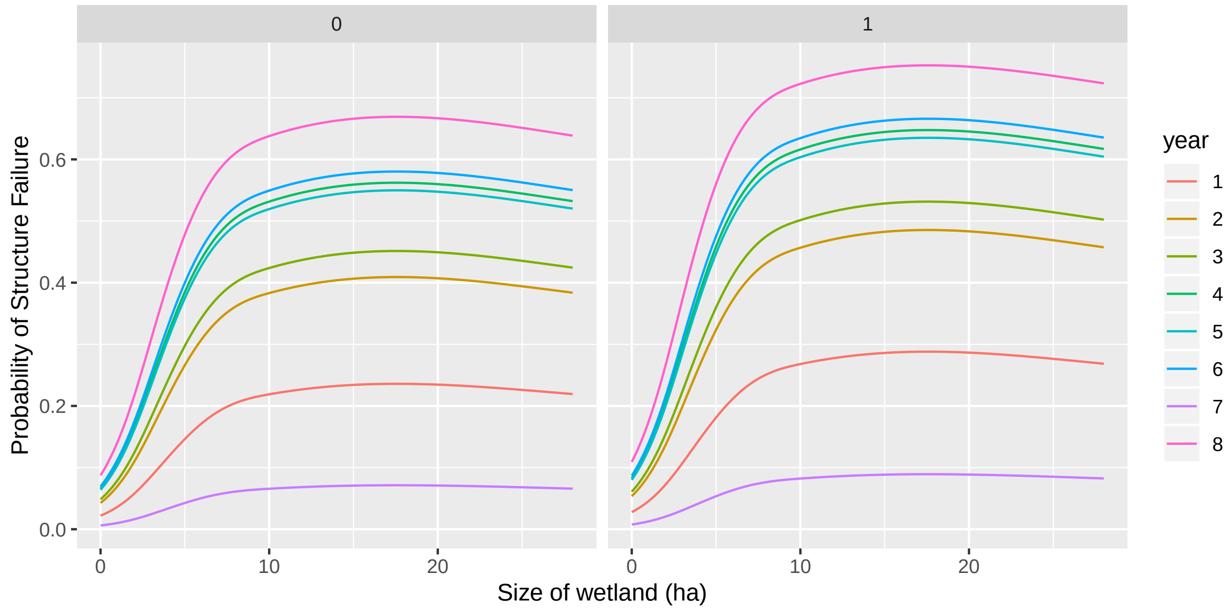

Get predictions averaged across years. We see that failure rates were slightly higher in double-cylinder than single-cylinder structures.

```
 pfail<- pdat2 %>% group_by(size, deply) %>% summarize(meanfail=mean(pfail))
 ggplot(pfail, aes(x=size, y=meanfail, color=deply))+geom_line() +
   xlab("Size of wetland (ha)")+ ylab("Probability of Structure Failure")
```

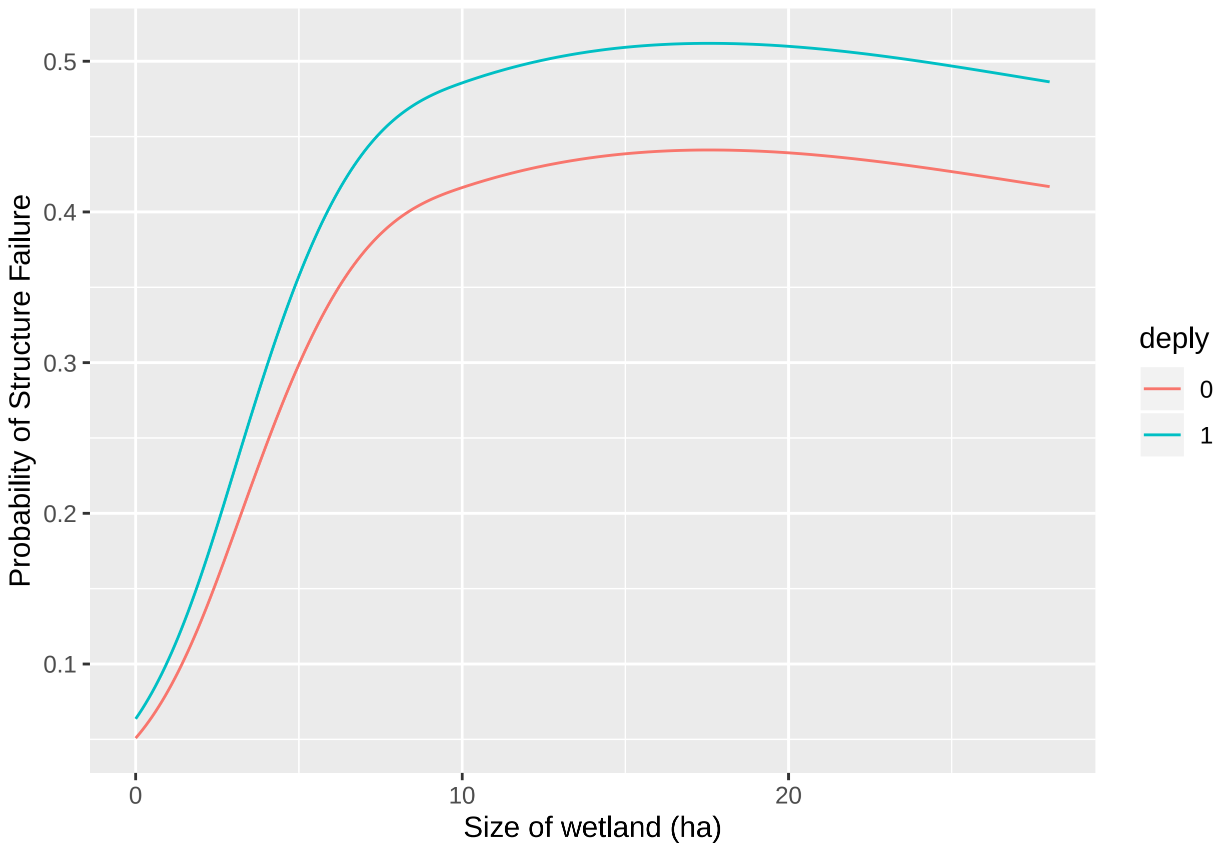

Costs were estimated as fixed ($25/cylinder + variable depending on survival and cylinder type)

- $28 if single cylinder fails, $48 if double cylinder fails

```
vcost<-ifelse(pfail$deply==0,28,48)
pfail$ec<-25+vcost*pfail$meanfail
ggplot(pfail, aes(x=size, y=ec, color=deply))+geom_line() +
  xlab("Size of wetland (ha)")+ ylab("Expected cost")
```

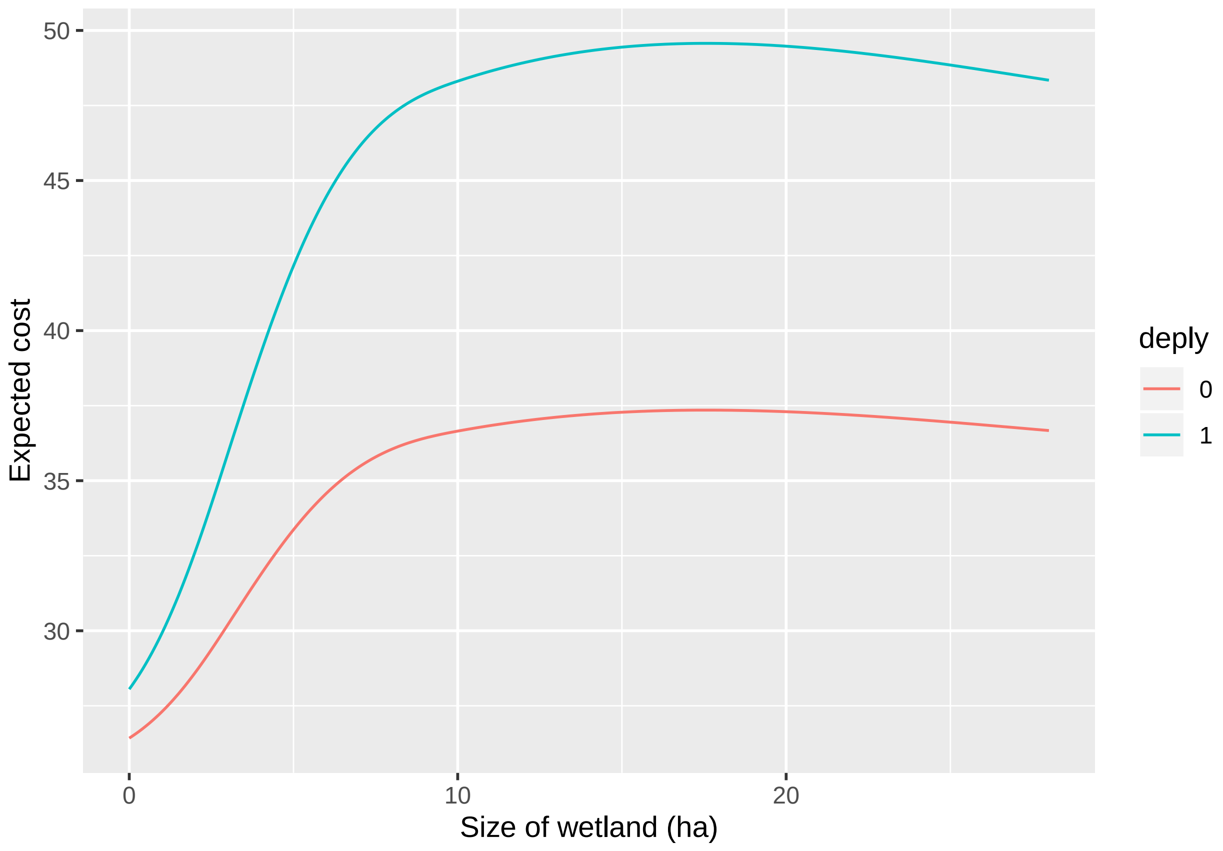

Duckling production model

Since the predictors do not change across years for this model, Zicus et al. just modeled the mean number of ducks as the response. Determine the mean

```
tdata<-ddata %>% group_by(strtno) %>% summarize(
  yng= mean(yng), size=mean(size), deply=unique(deply)
)
```

We can now model how the number of ducks depends on size of the wetland and cynlinder type

```
dmod<-lm(yng~size+I(size^2)+deply, data=tdata)
summary(dmod)
```

```
##
## Call:
## lm(formula = yng ~ size + I(size^2) + deply, data = tdata)
##
## Residuals:
##     Min      1Q  Median      3Q     Max
## -6.8467 -2.9481 -0.7668  2.4397 13.5074
##
## Coefficients:
##              Estimate Std. Error t value Pr(>|t|)
## (Intercept)  2.789003   0.570470   4.889 3.62e-06 ***
## size         0.361160   0.170658   2.116   0.0367 *
## I(size^2)   -0.008026   0.006873  -1.168   0.2455
```

```
## deply1       0.598900   0.715731   0.837   0.4046
## ---
## Signif. codes:  0 '***' 0.001 '**' 0.01 '*' 0.05 '.' 0.1 ' ' 1
##
## Residual standard error: 3.724 on 106 degrees of freedom
## Multiple R-squared:  0.1271, Adjusted R-squared:  0.1024
## F-statistic: 5.143 on 3 and 106 DF,  p-value: 0.002328
```

Note, the assumptions of linear regression are not met. In particular, the assumption that the residuals are Normally distributed seems suspect as evidenced by points falling off the line in the qqplot (top right). That's OK, we will use a non-parametric bootstrap for inference (resampling structures with replacement).

```
autoplot(dmod)
```

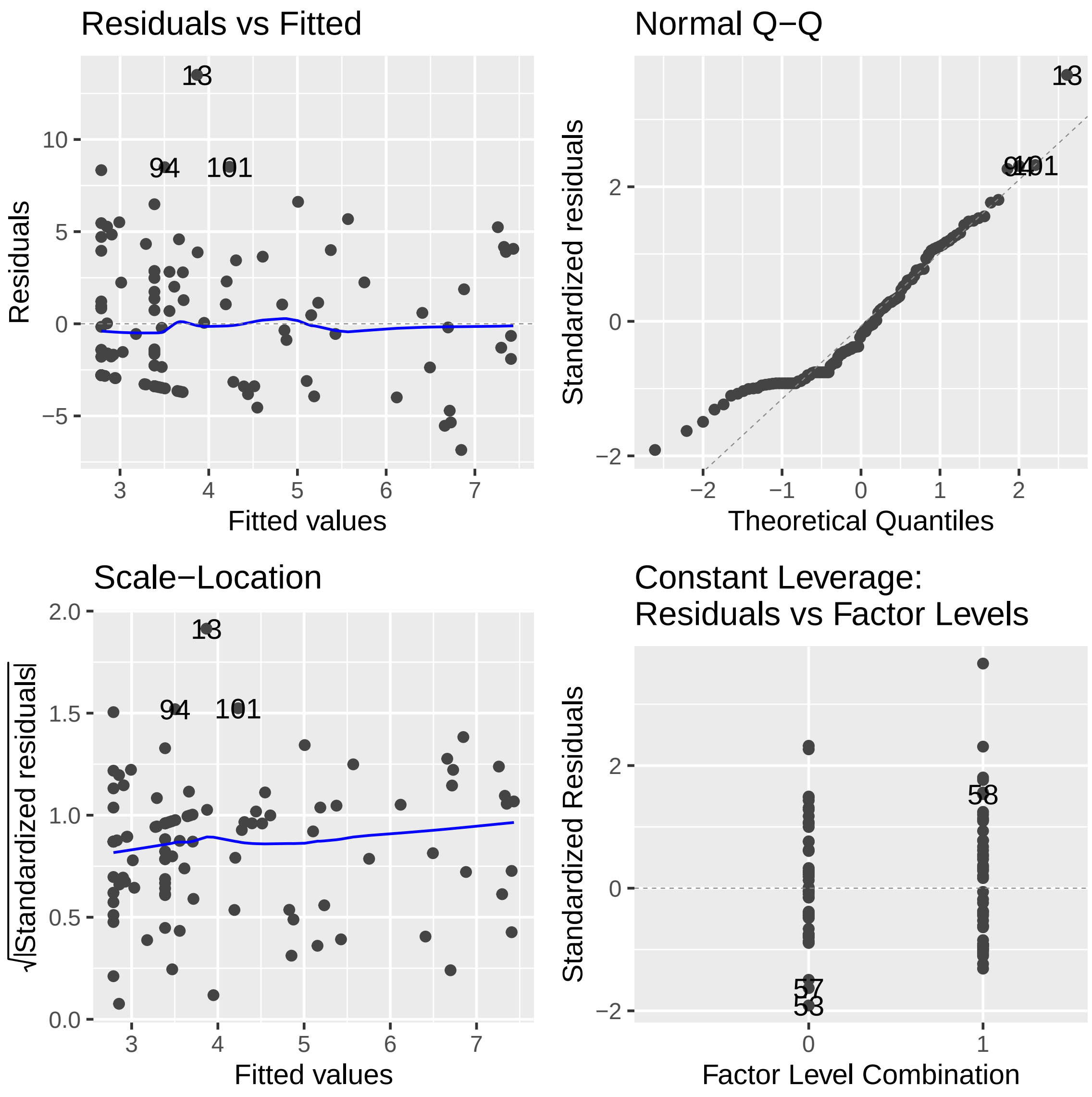

Predictions for the same range of wetland sizes and both deployment types

```
ducks<-expand.grid(size =seq(0,28,0.1), deply=unique(tdata$deply))
ducks$phat<-predict(dmod, newdata=ducks)
```

Order the ducks data set the same way as the structure failure data set

```
pfail
```

```
## # A tibble: 562 x 4
## # Groups:   size [281]
##     size deply meanfail    ec
##    <dbl> <fct>    <dbl> <dbl>
##  1   0   0       0.0508  26.4
##  2   0   1       0.0636  28.1
##  3   0.1 0       0.0534  26.5
##  4   0.1 1       0.0668  28.2
##  5   0.2 0       0.0561  26.6
##  6   0.2 1       0.0701  28.4
##  7   0.3 0       0.0589  26.6
##  8   0.3 1       0.0736  28.5
##  9   0.4 0       0.0618  26.7
## 10   0.4 1       0.0773  28.7
## # ... with 552 more rows
```

```
ducks<-ducks %>% arrange(size, deply)
head(ducks)
```

```
##   size deply     phat
## 1  0.0     0 2.789003
## 2  0.0     1 3.387903
## 3  0.1     0 2.825039
## 4  0.1     1 3.423939
## 5  0.2     0 2.860914
## 6  0.2     1 3.459814
```

Add plot showing expected ducks versus wetland size. Here, we see that larger wetlands may result in higher production rates of ducklings, but the data are highly variable and there is not much data available for structures placed in the largest wetlands.

```
  ggplot(ducks, aes(x=size, y=phat)) + geom_line() +
    geom_point(data=tdata, aes(size, y=yng))+ facet_wrap(~deply) +
    xlab("Size of wetland (ha)")+ ylab("Expected number of ducks")
```

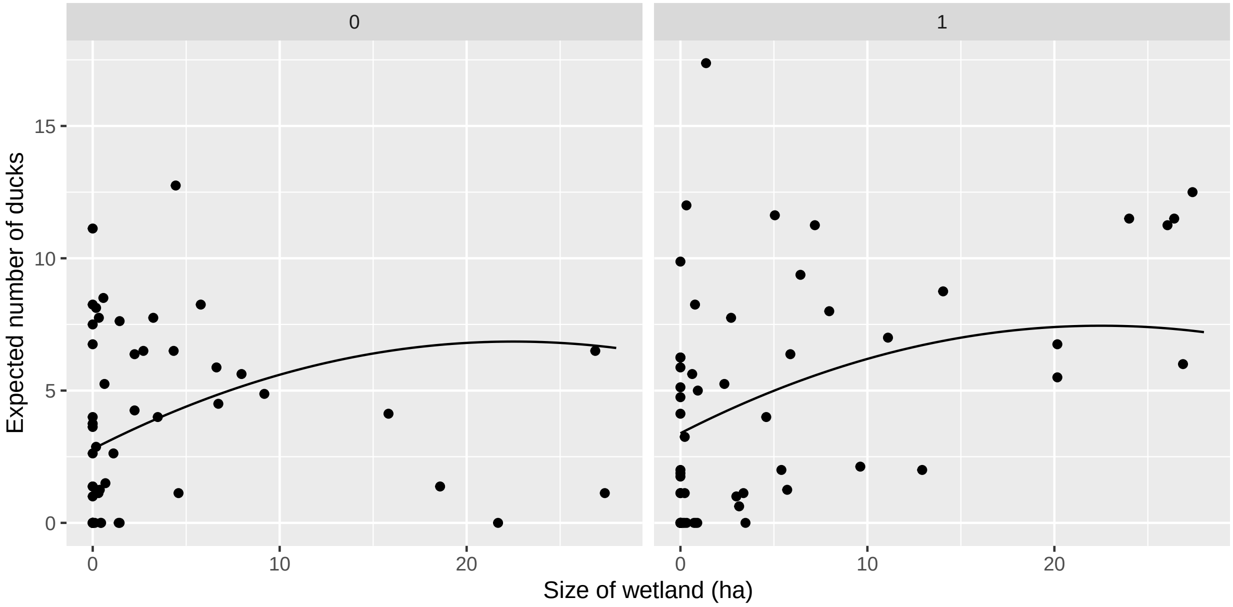

Get cost effectiveness by talking E[ducks]/E[cost]

```
costef<-inner_join(pfail, ducks)
```

```
## Joining, by = c("size", "deply")
```

```
costef$ce<- costef$ec/costef$phat
```

Plot cost-effectiveness versus wetland size for both cylinder types. We see that cost-effectiveness appears to be highest for deeper wetlands (due to higher duck production in these wetlands, despite higher failure probabilities associated with larger wetlands). However, we need to calculate a measure of uncertainty to help interpret these results.

```
ggplot(costef, aes(x=size, y=ce))+geom_line()+facet_wrap(~deply) +
  xlab("Size of wetland (ha)")+ ylab("Expected cost/expected ducks")
```

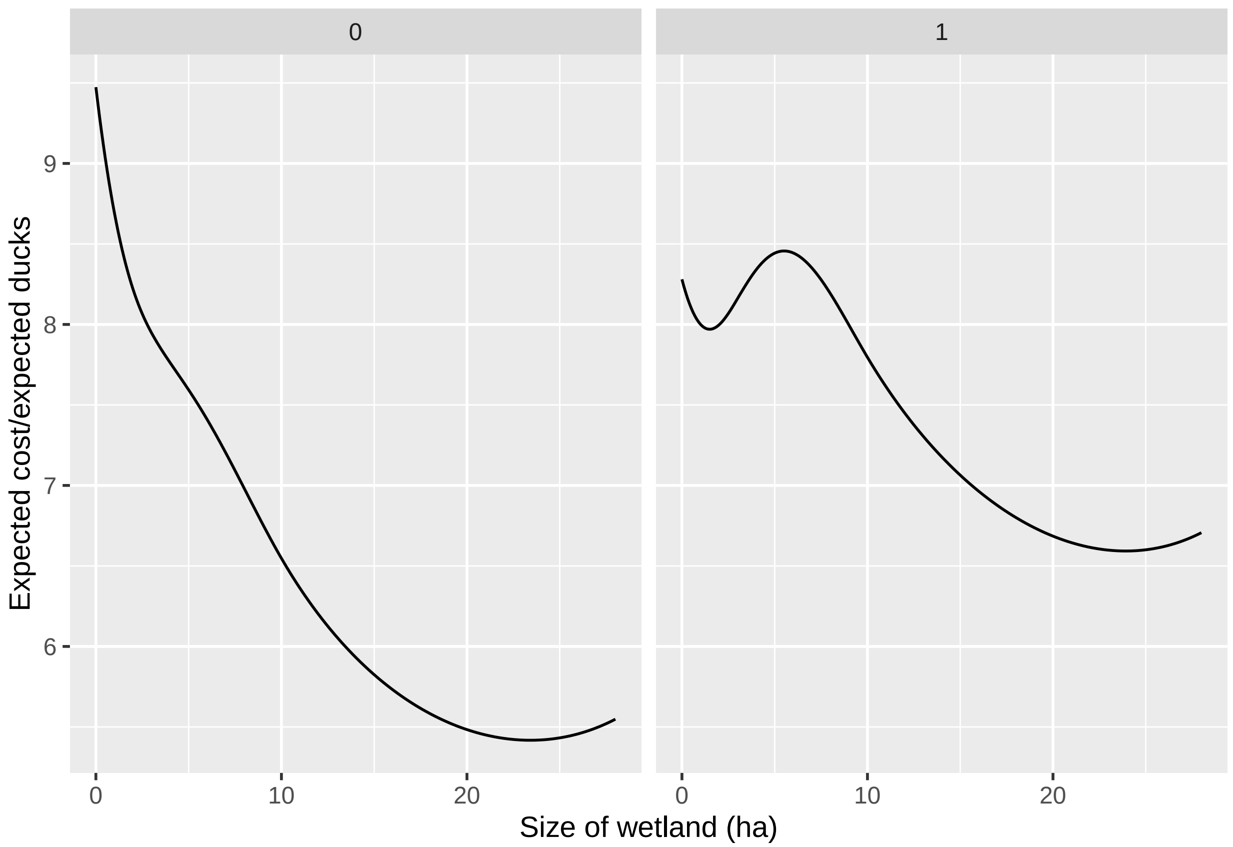

Cluster-level bootstrap to get estimates of uncertainty

Now that we have shown how to get an estimate of everything with the original data, to determine uncertainty, we just need to:

1. Resample structures with replacement.,
2. Refit our 2 models.
3. Recalculate our statistic of interest (ducks/expected cost) We will select structures with replacement

```
uid <- unique(ddata2$strtno) # unique id for each strtucture
nstrtno <- length(uid) # number of structures
```

Set up matrices to hold bootstrap results

```
nboot<-5000
pfail.b<-matrix(NA, nboot, nrow(ducks))
costs.b<-matrix(NA, nboot, nrow(ducks))
ducks.b<-matrix(NA, nboot, nrow(ducks))
pfail.temp<-pdat2
```

Code for the bootstrap is given below. This takes some time to run (and could be sped up by avoiding loops…).

```
for(i in 1:nboot){

  # Take a sample from x (uid) of size nstrtno with replacement:
  bootIDs <- data.frame(strtno = sample(x = uid, size = nstrtno, replace = TRUE))
```

```
    # Use this to sample from original data by beach
    bootDat <- merge(bootIDs,ddata2)

    # Now, fit survival model
    glmsurv.b<-glm(surv~year+deply +sz1+sz2+sz3, family=binomial(link=cloglog), data=bootDat)

    # Now, predictions on prob of failure (accounting for the
    # fact that our model is fit on the cloglog scale)
    pfail.temp$pfail<-predict(glmsurv.b, newdata=pdat2, type="resp")

    # Get predictions averaged across years
    pfail.temp2<- pfail.temp %>% group_by(size, deply) %>%
      summarize(meanfail=mean(pfail))
    pfail.b[i,]<-pfail.temp2$meanfail

    # Costs were estimated as fixed ($25/cylinder + variable depending on survival and cylinder type)
    costs.b[i,]<-25+vcost*pfail.b[i,]

    # ## Duckling production model

    # Since the predictors do not change across years for this model, Zicus et al. just
    # modeled the mean number of ducks as the response.  Determine the mean
    tdata.boot<-merge(bootIDs,tdata)

     # We can now model how the number of ducks depends on size
     # of the wetland and cynlinder type
    dmod.b<-lm(yng~size+I(size^2)+deply, data=tdata.boot)
    ducks.b[i,]<-predict(dmod.b, newdata=ducks)

}
```

Now, calculate pointwise 90% CI and plot.

```
  ducks$updducks<-apply(ducks.b,2, quantile, probs=0.95)
  ducks$lowducks<-apply(ducks.b,2, quantile, probs=0.05)
  pfail$upcost<-apply(costs.b, 2,quantile, probs=0.95)
  pfail$lowcost<-apply(costs.b, 2,quantile, probs=0.05)
  costef$upce<-apply(costs.b/ducks.b, 2, quantile, probs=0.95)
  costef$lowce<-apply(costs.b/ducks.b, 2, quantile, probs=0.05)
  costef$meanbootce<-apply(costs.b/ducks.b, 2, mean)
```

```
  ggplot(ducks, aes(x=size, y=phat)) +
    geom_ribbon(aes(ymin=lowducks, ymax=updducks), fill="grey70") +
    geom_line() + geom_point(data=tdata, aes(size, y=yng))+ facet_wrap(~deply) +
    xlab("Size of wetland (ha)")+ ylab("Expected number of ducks")
```

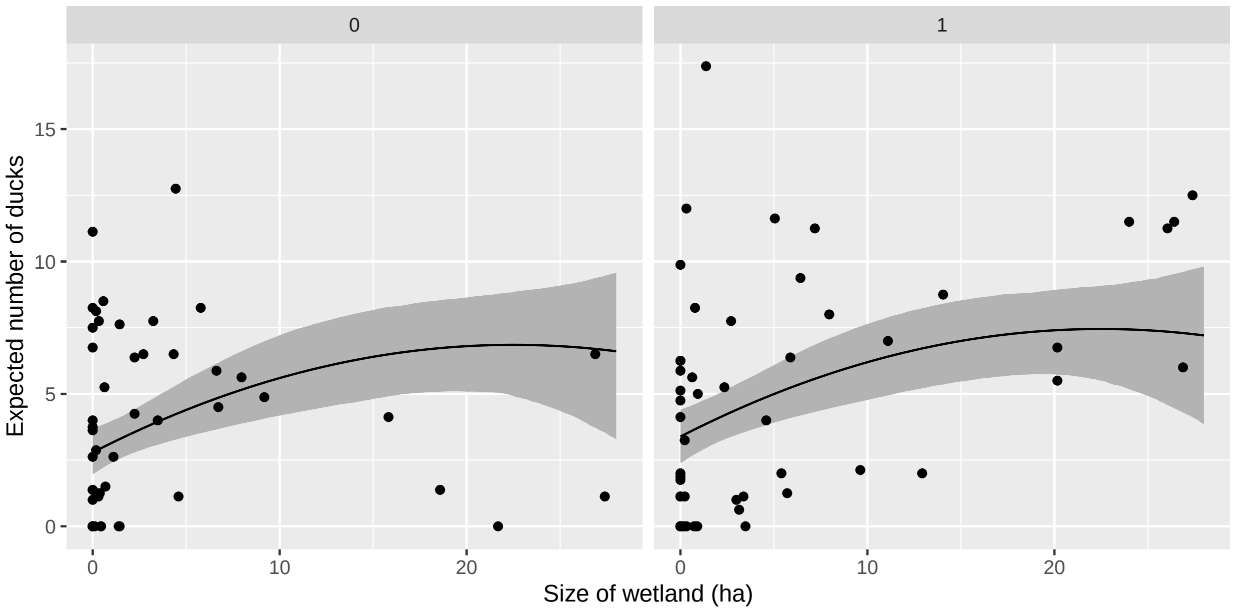


```
ggplot(pfail, aes(x=size, y=ec))+geom_ribbon(aes(ymin=lowcost, ymax=upcost), fill="grey70") +
  facet_wrap(~deply) + geom_line() +
  xlab("Size of wetland (ha)")+ ylab("Expected cost")
```

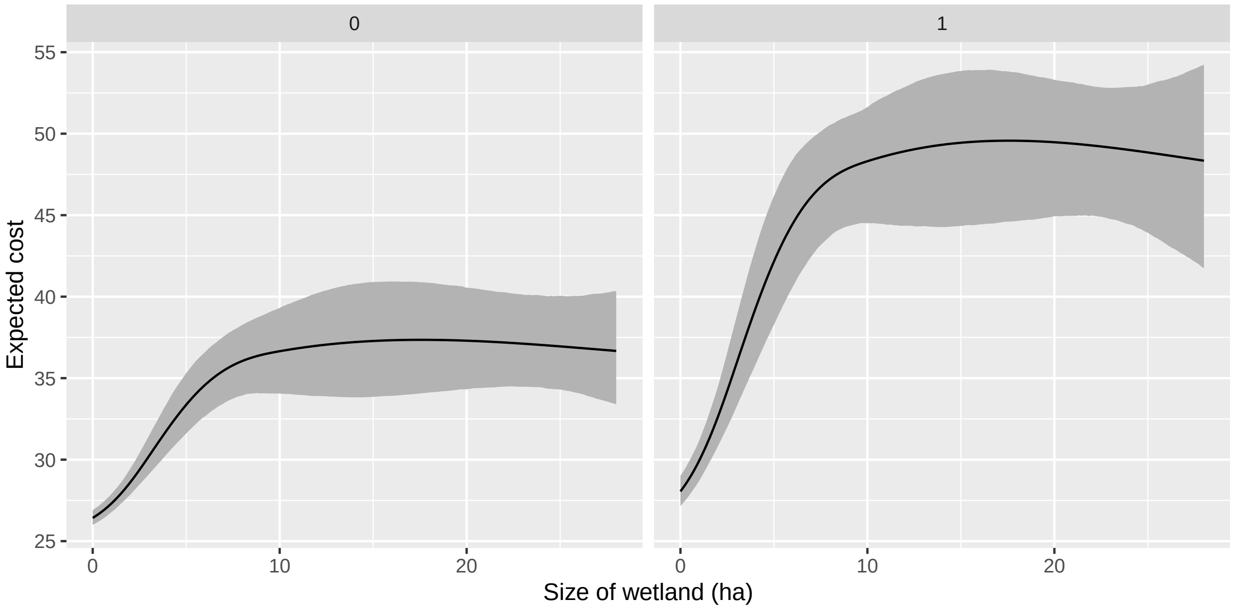


```
ggplot(costef, aes(x=size, y=ce))+geom_ribbon(aes(ymin=lowce, ymax=upce), fill="grey70") +
  facet_wrap(~deply) +geom_line()+
  xlab("Size of wetland (ha)")+ ylab("Expected cost/expected ducks")
```

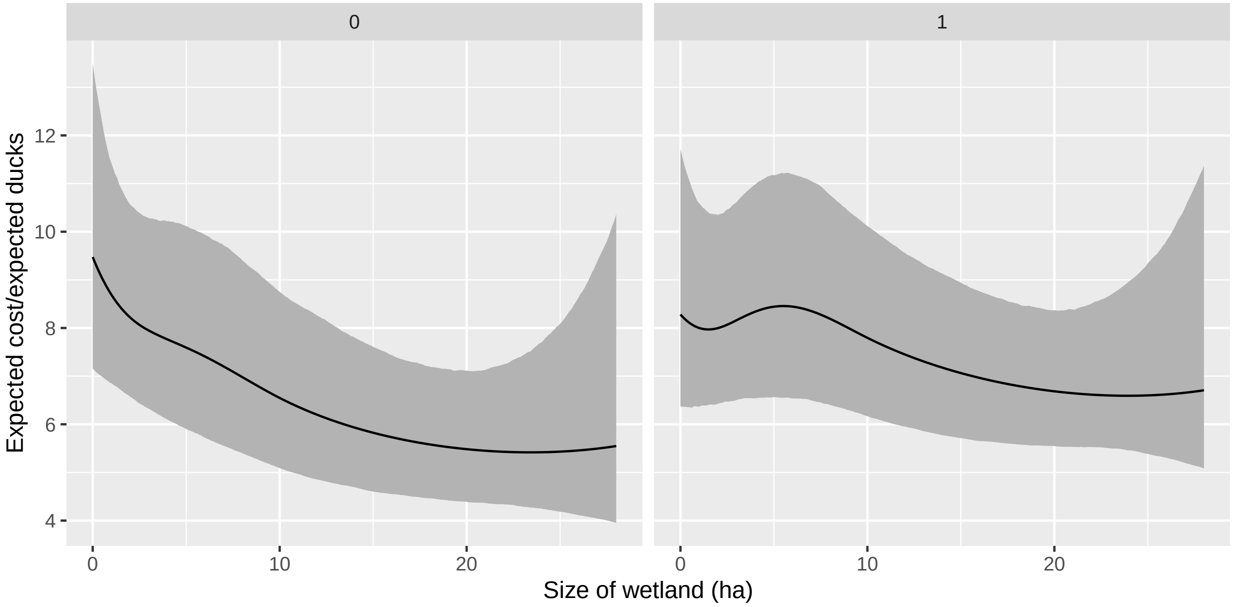

Use the bootstrap to check for bias

We can compare the mean of the bootstrap distribution to the point estimate as a measure of bias.

```
costef$boot.bias<-apply(costs.b/ducks.b, 2, mean) - costef$ce
```

Comparing the estimated bias to the estimated SE, we see that the relative bias is < 0.25, which Efron and Tibshirani (1993) suggest as a general rule of thumb for when it is not worth worrying about.

```
costef$boot.se<-apply(costs.b/ducks.b, 2, sd)
summary(costef$boot.bias/costef$boot.se)
```

```
##     Min.  1st Qu.   Median     Mean  3rd Qu.     Max.
## -0.01397  0.09027  0.12089  0.10756  0.12997  0.20730
```

Biological Conclusions

We conclude that cost-effectiveness of nest structures is highest in large wetlands (cost/duck is minimized). Cost-effectiveness also appears to be slightly higher for single-cylinder structures though confidence intervals for single- and double-cylinder structures largely overlap. Single-cylinder structures were less likely to fall over, so were less costly to maintain.

Document footer

Session Information:

```
  sessionInfo()
```

```
## R version 3.6.1 (2019-07-05)
## Platform: x86_64-w64-mingw32/x64 (64-bit)
## Running under: Windows 10 x64 (build 17763)
##
## Matrix products: default
##
## Random number generation:
##  RNG:     Mersenne-Twister
```

```
##  Normal:  Inversion
##  Sample:  Rounding
##
## locale:
## [1] LC_COLLATE=English_United States.1252
## [2] LC_CTYPE=English_United States.1252
## [3] LC_MONETARY=English_United States.1252
## [4] LC_NUMERIC=C
## [5] LC_TIME=English_United States.1252
##
## attached base packages:
## [1] splines   stats     graphics  grDevices utils     datasets  methods
## [8] base
##
## other attached packages:
## [1] ggfortify_0.4.7 ggplot2_3.2.1   dplyr_0.8.3     mgcv_1.8-28
## [5] nlme_3.1-140    gmodels_2.18.1  geepack_1.2-1
##
## loaded via a namespace (and not attached):
##  [1] ggrepel_0.8.1     Rcpp_1.0.2        lattice_0.20-38
##  [4] tidyr_1.0.0       gtools_3.8.1      utf8_1.1.4
##  [7] assertthat_0.2.1  zeallot_0.1.0     digest_0.6.22
## [10] packrat_0.5.0     mime_0.7          R6_2.4.0
## [13] backports_1.1.5   evaluate_0.14     ggstance_0.3.3
## [16] highr_0.8         pillar_1.4.2      rlang_0.4.1
## [19] lazyeval_0.2.2    rstudioapi_0.10   gdata_2.18.0
## [22] Matrix_1.2-17     rmarkdown_1.18    labeling_0.3
## [25] readr_1.3.1       stringr_1.4.0     htmlwidgets_1.5.1
## [28] munsell_0.5.0     shiny_1.4.0       broom_0.5.2
## [31] compiler_3.6.1    httpuv_1.5.2      xfun_0.10
## [34] pkgconfig_2.0.3   htmltools_0.4.0   tidyselect_0.2.5
## [37] tibble_2.1.3      gridExtra_2.3     mosaicCore_0.6.0
## [40] fansi_0.4.0       withr_2.1.2       crayon_1.3.4
## [43] later_1.0.0       MASS_7.3-51.4     grid_3.6.1
## [46] mosaicData_0.17.0 xtable_1.8-4      gtable_0.3.0
## [49] lifecycle_0.1.0   ggformula_0.9.2   magrittr_1.5
## [52] scales_1.0.0      cli_1.1.0         stringi_1.4.3
## [55] promises_1.1.0    leaflet_2.0.2     ggdendro_0.1-20
## [58] generics_0.0.2    vctrs_0.2.0       tools_3.6.1
## [61] glue_1.3.1        purrr_0.3.3       hms_0.5.2
## [64] crosstalk_1.0.0   fastmap_1.0.1     yaml_2.2.0
## [67] colorspace_1.4-1  mosaic_1.5.0      knitr_1.25
```
