# Supplementary material for: Resampling-based methods for biologists
Source: PeerJ. 2020 May 7;8:e9089. doi: 10.7717/peerj.9089 (PMC7211410; doi:10.7717/peerj.9089)
Supplement: Supplemental Information 4 — This example demonstrates how the bootstrap can be used to explore model uncertainty. [file peerj-08-9089-s004.pdf]

Case Study III: Model Selection Uncertainty

jfieberg

2020-01-09

Objective

This example demonstrates how the bootstrap can be used to explore model uncertainty.

Load R libraries

```
library(knitr)
library(rms)    # for validate function
library(MASS) # for stepAIC
```

Setting the seed of the random number generator

Use the **set.seed()** function in R to initialize the random number generator.

```
set.seed(2041971)
```

Modeling abundance of longnose dace

Read in the data:

```
dace<- read.csv("data/longnosedace.csv")
```

Predictors

- acreage = area (in acres) drained by the stream
- do2 = the dissolved oxygen (in mg/liter)
- depth = the maximum depth (in cm) of the 75-meter segment of stream
- no3 = nitrate concentration (mg/liter)
- so4 = sulfate concentration (mg/liter)
- temp = water temperature on the sampling date (in degrees C).

Fit a model using all 6 predictors, then use stepAIC to implement backwards selection to choose a "best" model.

```
fullmod.lm<-lm(longnosedace~acreage+do2+maxdepth+no3+so4+temp,data=dace)
stepAIC(fullmod.lm)
```

```
## Start:  AIC=511.82
## longnosedace ~ acreage + do2 + maxdepth + no3 + so4 + temp
##
##              Df Sum of Sq    RSS    AIC
## - so4         1       0.2 102787 509.82
## - do2         1    2165.8 104952 511.24
## <none>                    102787 511.82
## - temp        1    4432.8 107219 512.69
## - maxdepth    1    6638.2 109425 514.08
## - no3         1   11876.0 114663 517.26
## - acreage     1   14230.1 117017 518.64
##
## Step:  AIC=509.82
```

```
## longnosedace ~ acreage + do2 + maxdepth + no3 + temp
##
##            Df Sum of Sq    RSS    AIC
## - do2       1    2169.2 104956 509.24
## <none>                  102787 509.82
## - temp      1    4447.6 107234 510.70
## - maxdepth  1    6668.3 109455 512.10
## - no3       1   11935.8 114723 515.29
## - acreage   1   14268.0 117055 516.66
##
## Step:  AIC=509.24
## longnosedace ~ acreage + maxdepth + no3 + temp
##
##            Df Sum of Sq    RSS    AIC
## - temp      1    2948.0 107904 509.13
## <none>                  104956 509.24
## - maxdepth  1    6108.5 111064 511.09
## - acreage   1   14588.0 119544 516.09
## - no3       1   16501.4 121457 517.17
##
## Step:  AIC=509.13
## longnosedace ~ acreage + maxdepth + no3
##
##            Df Sum of Sq    RSS    AIC
## <none>                  107904 509.13
## - maxdepth  1    6058.4 113962 510.84
## - acreage   1   14652.0 122556 515.78
## - no3       1   16489.3 124393 516.80
```

```
##
## Call:
## lm(formula = longnosedace ~ acreage + maxdepth + no3, data = dace)
##
## Coefficients:
## (Intercept)      acreage     maxdepth          no3
##  -23.829067     0.001988     0.336605     8.673044
```

Bootstrap validation

Validate will use the bootstrap to calculate "honest" measures of model fit. We can also visualize "model uncertainty" in the "best model" by using bw=T (which tells R to use backwards selection to choose the best model). The "*" below indicate, which variables are included in the "optimal model" for each bootstrap replicate.

After applying a backwards model selection algorithm, we end up with a model containing only acreage and no3. The R^2 of this model = 0.24, which describes the variance in longnosedace explained by these two predictors. If we were to apply this same model to a new data set, we would expect the amount of variance that would be explained to be much lower. We can obtain a more "honest" measure of the variance by: a) creating 2 bootstrap data sets (one for model training and one for model testing); b) applying our model selection algorithm to the training data set and calculating the resulting R^2; c) use the same model to predict the response in the bootstrap test data set and use these predictions to calculate a second R^2; d) calculate a measure of "optimism" by subtracting the average R^2 from part c from the average R^2 in part b; e) subtract this estimate of optimism from the R^2 obtained from our original data set. The validate function will do this for us!

```r
fullmod.ols<-ols(longnosedace~acreage+do2+maxdepth+no3+so4+temp,data=dace, x=T, y=T)
validate(fullmod.ols, bw=T, B=100)
```

```
##
##       Backwards Step-down - Original Model
##
##  Deleted  Chi-Sq d.f. P      Residual d.f. P      AIC   R2
##  so4      0.00   1    0.9911 0.00     1    0.9911 -2.00 0.314
##  do2      1.29   1    0.2565 1.29     2    0.5253 -2.71 0.300
##  temp     1.75   1    0.1859 3.04     3    0.3860 -2.96 0.280
##  maxdepth 3.60   1    0.0579 6.63     4    0.1566 -1.37 0.239
##
## Approximate Estimates after Deleting Factors
##
##                 Coef      S.E.  Wald Z         P
## Intercept -2.861457 1.053e+01 -0.2717 0.7858203
## acreage    0.002325 6.502e-04  3.5754 0.0003497
## no3        9.012197 2.767e+00  3.2573 0.0011246
##
## Factors in Final Model
##
## [1] acreage no3
```

```
##           index.orig  training      test  optimism index.corrected   n
## R-square      0.2394    0.3383    0.1361    0.2022          0.0372 100
## MSE        1675.9166 1454.3732 1903.4649 -449.0918       2125.0084 100
## g            25.0102   29.4901   22.9498    6.5403         18.4698 100
## Intercept     0.0000    0.0000    7.6855   -7.6855          7.6855 100
## Slope         1.0000    1.0000    0.8254    0.1746          0.8254 100
##
## Factors Retained in Backwards Elimination
##
##  acreage do2 maxdepth no3 so4 temp
##              *                *
##              *                *
##  *           *        *
##  *       *   *        *   *   *
##          *   *                *
##  *                    *
##  *                    *
##  *       *            *       *
##  *       *            *   *
##  *                    *
##  *           *        *
##          *   *                *
##              *        *       *
##              *
##  *                    *
##  *                    *
##  *                    *       *
##              *        *
##  *       *   *        *
##              *        *       *
##  *                    *
```

```
##              *        *       *
##  *                    *       *
##  *           *                *
##  *                    *   *
##  *           *
##  *       *            *   *
##              *                *
##  *                    *
##  *                    *
##  *                    *
##  *           *        *
##              *                *
##  *                    *
##  *                    *
##  *           *        *       *
##  *           *        *       *
##  *       *                    *
##  *                    *
##  *                    *
##              *        *
##  *           *        *       *
##              *        *
##              *        *       *
##              *        *   *   *
##  *                    *
##  *                    *
##              *        *
##  *                            *
##  *                    *
##              *        *
##              *
##  *           *        *
##              *        *
##  *                    *
##  *                    *
##  *       *            *
##  *           *
##  *           *        *
##          *            *   *   *
##  *           *        *
##  *           *        *
##  *                    *
##  *                    *
##              *        *
##              *        *       *
##          *                *   *
##  *                    *       *
##  *
##  *           *        *       *
##  *           *        *
##  *       *   *
##  *       *   *        *       *
##  *                    *       *
##  *           *
```

```
##                       *       *
##  *                    *       *
##              *                *
##  *                    *
##          *   *
##              *                *
##                       *       *
##  *       *            *       *
##  *           *        *
##  *       *                    *
##  *                    *
##              *                *
##  *       *            *   *
##  *       *
##                       *
##  *       *   *                *
##          *   *
##  *                    *
##              *
##  *           *
##              *        *
##              *
##          *            *
##  *                    *
##  *       *            *
##
## Frequencies of Numbers of Factors Retained
##
##  1  2  3  4  5  6
##  6 50 29 13  1  1
```

Conclusions

1. We see that the different bootstrap samples result in different models being chosen as optimal. The number of predictor variables included ranges from 1 (in 6 models) to 6 (in 1 model).
2. We see that our original estimate of R^2 (0.24) is likely quite optimistic (our estimate of optimism = 0.20). Thus, we end up with a corrected estimate of $R^2 = 0.037$ (quite depressing!).

Footer

```r
# Session Information:
sessionInfo()
```

```
## R version 3.6.1 (2019-07-05)
## Platform: x86_64-w64-mingw32/x64 (64-bit)
## Running under: Windows 10 x64 (build 17763)
##
## Matrix products: default
##
## Random number generation:
##  RNG:     Mersenne-Twister
##  Normal:  Inversion
##  Sample:  Rounding
##
```

```
## locale:
## [1] LC_COLLATE=English_United States.1252
## [2] LC_CTYPE=English_United States.1252
## [3] LC_MONETARY=English_United States.1252
## [4] LC_NUMERIC=C
## [5] LC_TIME=English_United States.1252
##
## attached base packages:
## [1] splines   stats     graphics  grDevices utils     datasets  methods
## [8] base
##
## other attached packages:
##  [1] MASS_7.3-51.4     rms_5.1-3.1       SparseM_1.77
##  [4] Hmisc_4.2-0       Formula_1.2-3     survival_2.44-1.1
##  [7] mgcv_1.8-28       nlme_3.1-140      gmodels_2.18.1
## [10] geepack_1.2-1     boot_1.3-22       ggfortify_0.4.7
## [13] mosaic_1.5.0      Matrix_1.2-17     mosaicData_0.17.0
## [16] ggformula_0.9.2   ggstance_0.3.3    ggplot2_3.2.1
## [19] lattice_0.20-38   dplyr_0.8.3       knitr_1.25
##
## loaded via a namespace (and not attached):
##  [1] RColorBrewer_1.1-2  tools_3.6.1         backports_1.1.5
##  [4] utf8_1.1.4          R6_2.4.0            rpart_4.1-15
##  [7] lazyeval_0.2.2      colorspace_1.4-1    nnet_7.3-12
## [10] withr_2.1.2         tidyselect_0.2.5    gridExtra_2.3
## [13] leaflet_2.0.2       compiler_3.6.1      quantreg_5.51
## [16] cli_1.1.0           htmlTable_1.13.2    sandwich_2.5-1
## [19] ggdendro_0.1-20     labeling_0.3        mosaicCore_0.6.0
## [22] scales_1.0.0        checkmate_1.9.4     mvtnorm_1.0-11
## [25] polspline_1.1.16    readr_1.3.1         stringr_1.4.0
## [28] digest_0.6.22       foreign_0.8-71      rmarkdown_1.18
## [31] base64enc_0.1-3     pkgconfig_2.0.3     htmltools_0.4.0
## [34] fastmap_1.0.1       highr_0.8           htmlwidgets_1.5.1
## [37] rlang_0.4.1         rstudioapi_0.10     shiny_1.4.0
## [40] generics_0.0.2      zoo_1.8-6           crosstalk_1.0.0
## [43] gtools_3.8.1        acepack_1.4.1       magrittr_1.5
## [46] Rcpp_1.0.2          munsell_0.5.0       fansi_0.4.0
## [49] lifecycle_0.1.0     multcomp_1.4-10     stringi_1.4.3
## [52] yaml_2.2.0          grid_3.6.1          gdata_2.18.0
## [55] promises_1.1.0      ggrepel_0.8.1       crayon_1.3.4
## [58] hms_0.5.2           zeallot_0.1.0       pillar_1.4.2
## [61] codetools_0.2-16    glue_1.3.1          packrat_0.5.0
## [64] evaluate_0.14       latticeExtra_0.6-28 data.table_1.12.6
## [67] vctrs_0.2.0         httpuv_1.5.2        MatrixModels_0.4-1
## [70] gtable_0.3.0        purrr_0.3.3         tidyr_1.0.0
## [73] assertthat_0.2.1    xfun_0.10           mime_0.7
## [76] xtable_1.8-4        broom_0.5.2         later_1.0.0
## [79] tibble_2.1.3        tinytex_0.17        cluster_2.1.0
## [82] TH.data_1.0-10
```
